# Work Disability among Employees with Diabetes: Latent Class Analysis of Risk Factors in Three Prospective Cohort Studies

**DOI:** 10.1371/journal.pone.0143184

**Published:** 2015-11-16

**Authors:** Marianna Virtanen, Jussi Vahtera, Jenny Head, Rosemary Dray-Spira, Annaleena Okuloff, Adam G. Tabak, Marcel Goldberg, Jenni Ervasti, Markus Jokela, Archana Singh-Manoux, Jaana Pentti, Marie Zins, Mika Kivimäki

**Affiliations:** 1 Finnish Institute of Occupational Health, Helsinki, Turku and Tampere, Finland; 2 Department of Public Health, University of Turku and Turku University Hospital, Turku, Finland; 3 Department of Epidemiology and Public Health, University College London, London, United Kingdom; 4 INSERM, UMR_S 1136, Pierre Louis Institute of Epidemiology and Public Health, Department of Social Epidemiology, F-75013, Paris, France; 5 Sorbonne Universités, UPMC Univ Paris 06, UMR_S 1136, Pierre Louis Institute of Epidemiology and Public Health, Department of Social Epidemiology, F-75013, Paris, France; 6 1st Department of Medicine, Faculty of Medicine, Semmelweis University, Budapest, Hungary; 7 Population-based Cohorts Unit, Inserm UMS 011, Villejuif, France; 8 University Versailles Saint Quentin en Yvelines, Versailles, France; 9 Institute of Behavioral Sciences, University of Helsinki, Helsinki, Finland; 10 Inserm U1018, Centre for Research in Epidemiology and Population Health, Villejuif, France; 11 Clinicum, Faculty of Medicine, University of Helsinki, Helsinki, Finland; Universidad Peruana Cayetano Heredia, PERU

## Abstract

**Background:**

Studies of work disability in diabetes have examined diabetes as a homogeneous disease. We sought to identify subgroups among persons with diabetes based on potential risk factors for work disability.

**Methods:**

Participants were 2,445 employees with diabetes from three prospective cohorts (the Finnish Public Sector study, the GAZEL study, and the Whitehall II study). Work disability was ascertained via linkage to registers of sickness absence and disability pensions during a follow-up of 4 years. Study-specific latent class analysis was used to identify subgroups according to prevalent comorbid disease and health-risk behaviours. Study-specific associations with work disability at follow-up were pooled using fixed-effects meta-analysis.

**Results:**

Separate latent class analyses for men and women in each cohort supported a two-class solution with one subgroup (total n = 1,086; 44.4%) having high prevalence of chronic somatic diseases, psychological symptoms, obesity, physical inactivity and abstinence from alcohol and the other subgroup (total n = 1,359; 55.6%) low prevalence of these factors. In the adjusted meta-analyses, participants in the ‘high-risk’ group had more work disability days (pooled rate ratio = 1.66, 95% CI 1.38–1.99) and more work disability episodes (pooled rate ratio = 1.33, 95% CI 1.21–1.46). These associations were similar in men and women, younger and older participants, and across occupational groups.

**Conclusions:**

Diabetes is not a homogeneous disease in terms of work disability risk. Approximately half of people with diabetes are assigned to a subgroup characterised by clustering of comorbid health conditions, obesity, physical inactivity, abstinence of alcohol, and associated high risk of work disability; the other half to a subgroup characterised by a more favourable risk profile.

## Introduction

Worldwide, more than 340 million people have diabetes [1] and years lived with disability due to diabetes has almost doubled between 1990 and 2010 [2]. As diabetes is also associated with reduced productivity, working capacity, and increased disability [3–5] it is important to identify factors that might help reduce disease complications, such as work disability, among individuals with diabetes.

While the adverse impact of diabetes on morbidity and mortality have been widely reported [6], less is known about work disability associated with diabetes. A recent systematic review identified 8 studies on diabetes and absenteeism, of which only 3 were prospective cohort studies [4]. The majority of studies reported higher sickness absenteeism among people with diabetes compared to those without diabetes. Importantly, previous research has examined persons with diabetes as a single group without taking into account possible heterogeneity of the population with diabetes. For example, major risk factors of work disability—comorbid chronic diseases, obesity, physical inactivity, smoking, and high alcohol use [7–13]–may cause significant heterogeneity in the risk of work disability among people with diabetes. Latent class analysis (LCA) [14] is a statistical tool to determine naturally occurring subgroups within patient populations, but no such studies have been undertaken in relation to diabetes and work disability.

In the present study of participants diagnosed with diabetes in three cohort studies, we first analysed the association between each potential risk factor and work disability separately. We then applied LCA to investigate heterogeneity in the population with diabetes and to identify possible subgroups based on potential risk factors for disability. As the final step, the degree to which these subgroups had different risk of future work disability was examined.

## Methods

We used data from three European cohort studies, the Finnish Public Sector study (FPSS) from Finland, the GAZEL study from France and the Whitehall II study from the United Kingdom.

### Ethics Statement

In the FPSS, the Ethics Committee of the Hospital District of Helsinki and Uusimaa approved the study; the response to a questionnaire acted as a form of written informed consent. The GAZEL study was approved by the Inserm Ethics committee and all participants gave written informed consent to participate. Ethical approval for the Whitehall II study was obtained from the University College London Medical School committee on the ethics of human research; all participants provided written informed consent. All data were analyzed anonymously.

### Study Populations

FPSS concerns workers of 10 towns and 21 hospitals [10, 15]. For the present study, the base cohort was participants who responded to the survey in 2004 (*n* = 48,076, response rate 66%). Of the respondents, 1,359 were identified as having diabetes. After exclusion of people who died or retired during one year after baseline, the analytic sample was 1,324 (368 men, 956 women).

The GAZEL cohort study, established in 1989, is comprised of employees from the French national gas and electricity company: Electricité de France-Gaz de France (EDF-GDF) [16]. At baseline, 20,625 employees (73% men), aged 35–50 years, participated. Of the 20,625 participants who responded to at least one survey between the years 1989 and 2003, 914 were identified as having diabetes. After exclusion of people who died or retired during one year after baseline, the analytic sample was 842 (678 men, 164 women).

The Whitehall II study is a prospective cohort study of British civil servants (government employees) [17]. The target population was all London-based office staff, aged 35–55 years, working in 20 civil service departments on recruitment to the study in 1985–88. At baseline 73% (10,308 employees) participated. Since then, 8 follow-up examinations have taken place approximately every 2 to 3 years. Diabetes cases were identified from 1985 to the end of 1997 (study phases 1–5), for which data on sickness absence for follow-up were available. We detected 279 cases of diabetes who had at least one year of follow-up time (196 men, 83 women).

### Ascertainment of Diabetes

In FPSS, identification of diabetes cases was based on three sources: (1) national registers of purchased diabetes medicines (oral medication or insulin); (2) entitlements to special reimbursements for the costs of medication by the Social Insurance Institution of Finland; and 3) from responses to a survey question on doctor-diagnosed diabetes. In GAZEL, diabetes was identified from responses to a checklist of over 50 doctor-diagnosed chronic conditions in annual surveys. In Whitehall II diabetes was assessed by self-reported doctor-diagnosed diabetes, or use of diabetes medication (all phases) and this was supplemented by the results of fasting glucose and a two-hour oral glucose tolerance tests in study phases 3 and 5. Diabetes was diagnosed by fasting glucose of ≥7.0 mmol/L or oral glucose tolerance test (2-hour post-load glucose ≥11.1 mmol/L) [18].

### Risk Factors

In all cohorts, comorbid conditions included cardiovascular disease, hypertension, asthma, rheumatoid arthritis and depression or psychological symptoms. In FPSS, data on comorbid physical diseases were based on entitlements to special reimbursement, and data on psychological symptoms was based on the General Health Questionnaire (GHQ) 12-item version [19]. In GAZEL, data for all comorbid diseases were derived from survey responses to a checklist of doctor-diagnosed chronic conditions. In Whitehall II, information on coronary heart disease was based on clinical examination, medical records, and hospital records. Information on comorbid physical diseases was based on self-reported longstanding illnesses, and information on psychological symptoms was based on the GHQ 30-item version [19, 20]. In FPSS and GAZEL, self-reported height and weight and in Whitehall II, height and weight measured at the study clinic were used to calculate body mass index (BMI) from which overweight (BMI = 25–29) and obese (BMI≥30 kg/m^2^) participants were identified. Low physical activity was measured through the harmonized measure of the large European-wide Iindividual-Participant-Data on Working Conditions study [21]. Participants who reported none or very little moderate or vigorous leisure-time physical activity or exercise were coded as having low physical activity. Smoking was categorized as current smoker versus non-smoker in all cohorts. Average use of beer, wine and spirits consumed per week (FPSS, Whitehall II) or day (GAZEL) was requested and transformed into units of alcohol per week. Risky alcohol use was defined as ≥22 units/week (men) or ≥15 units/week (women) [22].

### Work Disability Outcomes

In the FPSS, data on work disability were derived from registers of the Social Insurance Institution of Finland (sickness absence) and the Finnish Centre for Pensions (fixed-term and permanent work disability pensions). Absences were tracked from 2005 to 2009 (mean follow-up 4.7, SD = 0.9 years). In GAZEL, data on sickness absence and disability pensions were obtained from employer records from 1990 to 2009 (mean follow-up 3.9, SD = 1.5 years) and absences were tracked from the first occurrence of self-reported diabetes. In Whitehall II, information on sickness absence was obtained from Civil Service (employer) records from January 1986 to 1998 and data on retirement on health grounds from survey responses (mean follow-up 4.6, SD = 3.6 years). In all cohorts, shorter follow-up was due to old-age retirement or death and in Whitehall II and GAZEL, also due to leaving the organization. In FPSS the follow-up time for all participants begun in Jan 1, 2005 after the baseline survey in 2004. In GAZEL and Whitehall II the follow-up time begun from the phase when diabetes was first detected, and risk factors and covariates were derived from the survey at that phase or if data were not available from the most recent survey. We used two outcome variables: (1) absence duration, as measured by the number of absence days (named ‘work disability days’) and (2) absence frequency (‘work disability episodes’). Both outcomes were based on sickness absences and disability pensions. An episode of work disability could include a period of sickness absence, or temporary or permanent disability pension award.

### Covariates

Socio-demographic baseline covariates were age, sex, occupational grade (based on occupational grade and categorised as high, intermediate and low), and marital status (married or cohabiting versus single, divorced or widowed). Data on occupations in each cohort were based on company records (FPSS and GAZEL) or self-reports (Whitehall II). In FPSS, high occupational grade included e.g., teachers and physicians, intermediate grade e.g., registered nurses and technicians, and low grade included e.g., cleaners and maintenance workers [15]. The GAZEL study comprises employees in executive and middle management positions (high grade), other non-manual workers (intermediate grade) and manual workers (low grade) [16]. In Whitehall II, the corresponding occupational groups were administrative (high grade), professional or executive (intermediate grade), and clerical or support (low grade) [23]. These indicators of socioeconomic status have previously been used in the Individual-Participant-Data Meta-analysis in Working Populations project [24]. For analyses, occupational grade was dichotomised as intermediate/low versus high.

### Statistical Analysis

We used negative binomial regression analysis (models that are designed to analyse count data) to assess rate ratios (RR) and their 95% confidence intervals (CI) for the single associations of sociodemographic factors, comorbidity and risk factors at baseline with work disability outcomes at follow-up (i.e., the number of work disability days and episodes) in each cohort. The models were adjusted for age, sex, occupational grade and marital status. We pooled the study-specific results using fixed-effects meta-analysis and examined heterogeneity of the study-specific estimates using the *I* ^*2*^ statistics.

We conducted latent lass analysis (LCA) [14] in each cohort using sex as a group factor to identify subgroups according to prevalent comorbid disease, psychological symptoms, obesity, physical inactivity, smoking and alcohol use. LCA is a statistical tool which clusters similar response profiles into distinct classes. We fitted models with up to 9 classes and selected models with the best fit assessed using the Akaike information criterion (AIC), consistent AIC, Bayesian information criterion (BIC), adjusted BIC, and entropy [25–29] where the models with the smallest values indicate a better fit. Individuals were then assigned to their most likely class. We used logistic regression analysis to examine the association of socio-demographic characteristics with the likelihood for membership to a specific latent class (the ‘high-risk profile’ subgroup).

We used negative binomial regression to assess RR with their 95% CIs for the associations of ‘high-risk profile’ membership and the risk of work disability days and episodes, adjusted for age, sex, occupational grade, and marital status, among all participants and separately for men and women and age and occupational groups in each cohort study. The study-specific estimates were pooled using fixed-effects meta-analysis. Meta-regression analysis was used to assess subgroup differences. All study-specific analyses were performed using SAS 9.4 (PROC GENMOD and PROC LCA procedures) statistical software and meta-analyses were performed using fixed-effects meta-analysis in Stata (METAN procedure) version 13. Level of P<0.05 was used to define statistical significance.

## Results

Descriptive characteristics of the study participants by sex and cohort are shown in Table 1. Any differences in age at baseline between the cohorts were relatively small. Men in FPSS and women in GAZEL and Whitehall II were more often from the low occupational grade group. Men in GAZEL were more often and women in Whitehall II less often married or cohabiting than men and women in other cohorts. Men in GAZEL had a lower proportion and women in GAZEL had a higher proportion of comorbid somatic diseases than participants in other cohorts and FPSS women had a relatively high prevalence of psychological symptoms. A relatively low prevalence of obesity was found among Whitehall II men and relatively high prevalence of those with physical inactivity was found among GAZEL women. Highest number of smoking participants was found among GAZEL men and those with high alcohol use was found among FPSS and GAZEL men. Most abstinent participants were found among women in GAZEL and Whitehall II. Whitehall II women had the highest level and GAZEL men the lowest level of work disability days. Whitehall II women also had the highest level of work disability episodes while FPSS men had the lowest level of episodes.

**Table 1:** Characteristics of men and women in three study cohorts.

	Finnish Public Sector study		GAZEL study		Whitehall II study	
Characteristic	Men (*n* = 368)	Women (*n* = 956)	Men (*n* = 678)	Women (*n* = 164)	Men (*n* = 196)	Women (*n* = 83)
Age (mean, SD)	51.3 (8.1)	47.4 (9.4)	49.2 (3.9)	48.5 (5.0)	49.9 (6.6)	49.7 (6.1)
Occupational grade (*n*, %)						
High	112 (30.4)	221 (23.1)	252 (37.2)	21 (12.9)	52 (26.5)	6 (7.2)
Intermediate	114 (31.0)	563 (59.0)	332 (49.0)	107 (65.6)	113 (57.7)	29 (34.9)
Low	142 (38.6)	171 (17.9)	94 (13.9)	35 (21.5)	31 (15.8)	48 (57.8)
Married / cohabited (*n*, %)						
Yes	285 (79.0)	694 (73.2)	604 (89.2)	116 (70.7)	149 (78.0)	48 (60.0)
No	76 (21.1)	254 (26.8)	73 (10.8)	48 (29.3)	42 (22.0)	32 (40.0)
Comorbid somatic disease (*n*, %)						
No	202 (54.9)	627 (65.6)	397 (59.8)	69 (42.9)	115 (58.7)	53 (63.9)
Yes	166 (45.1)	329 (34.4)	267 (40.2)	92 (57.1)	81 (41.3)	30 (36.1)
Psychological symptoms (*n*, %*)*						
No	263 (72.5)	654 (68.8)	632 (93.2)	127 (77.4)	148 (78.3)	57 (72.2)
Yes	100 (27.6)	297 (31.2)	46 (6.8)	37 (22.6)	41 (21.7)	22 (27.9)
Body mass index (BMI) (*n*, %)						
<25	80 (22.1)	268 (29.3)	188 (29.0)	69 (44.5)	78 (41.9)	33 (40.7)
25–29	150 (41.4)	328 (35.9)	315 (48.6)	41 (26.5)	79 (42.5)	22 (27.2)
≥30	132 (36.5)	318 (34.8)	145 (22.4)	45 (29.0)	29 (15.6)	26 (32.1)
Low physical activity (*n*, %)						
No	235 (64.6)	657 (69.2)	363 (58.5)	72 (49.0)	132 (69.8)	47 (61.0)
Yes	129 (35.4)	292 (30.8)	258 (41.6)	75 (51.0)	57 (30.2)	30 (39.0)
Smoking (*n*, %)						
No	269 (77.1)	763 (82.5)	514 (75.9)	131 (80.4)	150 (79.8)	72 (88.9)
Yes	80 (22.9)	162 (17.5)	163 (24.1)	32 (19.6)	38 (20.2)	9 (11.1)
Alcohol use (*n*, %)						
No	39 (10.7)	195 (20.6)	65 (10.8)	51 (34.0)	42 (25.3)	40 (51.3)
Moderate	236 (64.8)	677 (71.4)	383 (63.6)	80 (53.3)	112 (67.5)	36 (46.2)
High	89 (24.5)	76 (8.0)	154 (25.6)	19 (12.7)	12 (7.2)	2 (2.6)
Study baseline year(s)	2004	2004	1989–2003	1989–2003	1985–1997	1985–1997
Years of follow-up (mean, SD)	4.6 (1.0)	4.7 (0.8)	3.9 (1.5)	4.2 (1.4)	4.3 (3.5)	5.4 (3.6)
Work disability days during follow-up (mean, SD) / mean/year	176.8 (380.4) / 38.4	169.4 (348.5) / 36.0	65.4 (163.4) / 16.8	106.5 (222.3) / 25.4	119.3 (406.5) / 27.7	249.9 (581.5) / 46.3
Work disability episodes during follow-up (mean, SD) / mean/year	1.3 (1.6) / 0.3	1.7 (1.9) / 0.4	2.7 (3.9) / 0.7	5.3 (5.6) / 1.3	8.5 (12.5) / 2.0	12.2 (11.8) / 2.3
No. of work disability days / 100 person-years	3885.8	3603.0	1686.0	2539.1	2787.6	4657.8
No. of work disability episodes / 100 person-years	28.4	36.3	70.4	125.1	198.4	228.1

Note. Cell frequencies may vary due to missing data.

S1 Fig displays pooled data assessing risk factors for work disability outcomes individually. We found that female sex, age ≥50 years, lower occupational grade, comorbid somatic disease, psychological symptoms, obesity, low physical activity, and abstinence from alcohol at baseline were all associated with higher number of work disability days at follow-up. Similarly, women, those with lower occupational grade, comorbid somatic disease, psychological symptoms, obesity, low physical activity, and abstinence from alcohol were more likely to have a higher number of work disability episodes. Heterogeneity between study cohorts in the study-specific estimates was significant for the association of age (I^2^ = 91.5%, p<0.001), comorbid somatic disease (I^2^ = 85.3%, p = 0.001), and smoking (I^2^ = 73.4%, p = 0.023) with work disability days, as well as the association of sex (I^2^ = 73.9%, p = 0.022), age (I^2^ = 69.6%, p = 0.037), and comorbid somatic disease (I^2^ = 81.0%, p = 0.005) with work disability episodes (data not shown in the figure). To assess whether the somewhat surprising association between abstinence from alcohol and work disability was owing to confounding factors, we further adjusted the models for comorbid somatic disease, psychological symptoms, BMI, and physical activity. The association with work disability days (RR = 1.51, 95% CI 1.19–1.91), and episodes (RR = 1.15, 95% CI 1.02–1.31) slightly attenuated but remained statistically significant after further adjustment for these potential confounding factors.

In the latent class analysis, a two-class solution yielded the best fit in each cohort (S1 Table). Similar results were obtained among men and women (data not shown in the table).

**Fig 1:**
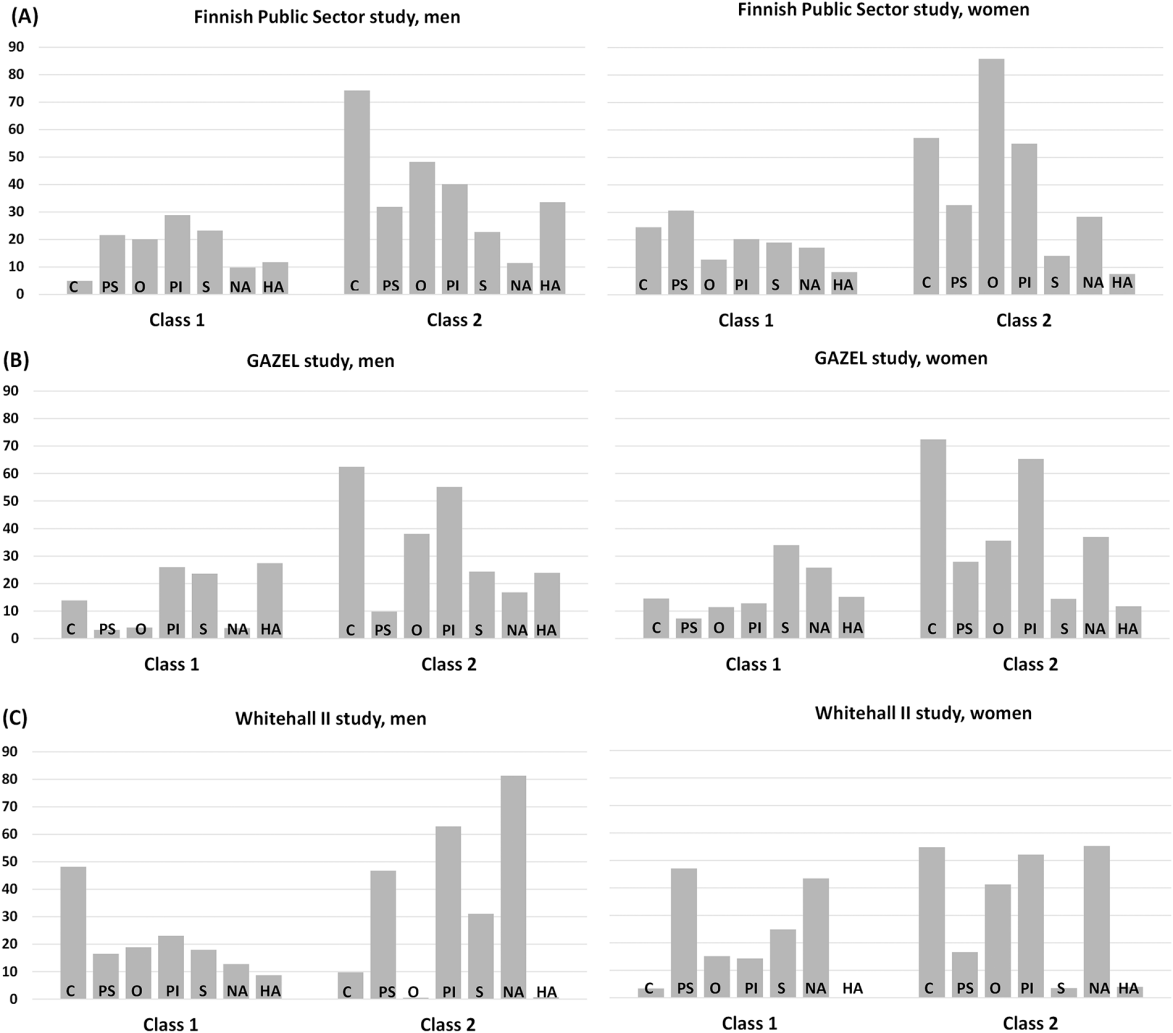
Class membership and risk factor probabilities across latent classes among men and women in each cohort (panels A to C; class 1 = ‘low-risk profile’ and class 2 = ‘high-risk profile’). Footnote: C = Comorbid somatic disease; PS = Psychological symptoms; O = Obesity; PI = Physical inactivity; S = Smoking; NA = No alcohol use; HA = High alcohol use.

As shown in Fig 1, class 1 was mostly characterised by lower prevalence of comorbid somatic disease, physical inactivity, obesity and abstinence from alcohol, as well as lower prevalence of psychological symptoms than that seen in the total cohort. Class 2, denoting to a ‘high-risk profile’ subgroup was characterized by higher prevalence of these factors—which were all also risk factors of work disability in our previous analysis of single risk factors. However, there were some exceptions; in Whitehall II men, comorbid somatic disease and in Whitehall II women, psychological symptoms were more likely to be prevalent in class 1 than class 2. In addition, high alcohol use and smoking did not differentiate members of the two classes; high alcohol use was more prevalent in class 1 (Whitehall II men), equally prevalent in the two classes (FPSS and Whitehall II women, GAZEL men and women), or more prevalent in class 2 (FPSS men). Smoking was more prevalent in class 1 (FPSS, GAZEL and Whitehall II women), equal prevalent in the two classes (FPSS and GAZEL men), or more prevalent in class 2 (Whitehall II men).

Overall 55.6% (42.0% of men and 69.6% of women) were assigned to class 1 (‘low-risk profile’ subgroup), and respectively, 44.4% (58.0% of men and 30.4% of women) were assigned to class 2 (‘high-risk profile’ subgroup) although some variation was found between cohorts. In the FPSS, 58.0% of men and 30.4% of women were assigned to class 2. The corresponding percentages were 54.3% for men and 73.6% for women in GAZEL, and 17.7% for men and 64.0% for women in Whitehall II (data not tabulated).

Table 2 shows associations between sociodemographic factors and the likelihood of belonging to the ‘high-risk profile’ subgroup (class 2). Of the sociodemographic factors, age ≥50 years (pooled odds ratio, OR = 1.64) and lower occupational grade (pooled OR = 1.60) were associated with membership of ‘high-risk profile’ whereas marital status was not. There was some heterogeneity between study cohorts in the association of age among women (I^2^ = 69.4%, P-value = 0.04). However, meta-regression analyses did not suggest significant difference between age, occupational grade, or marital status groups in the association with membership of the ‘high-risk profile’ subgroup (P-values ≥0.80).

**Table 2:** Association between socio-demographic factors and likelihood of being in a ‘high-risk profile’ subgroup among men and women with diabetes.

	**Men**					
**Characteristic**	**FPSS (*n* = 361)**		**GAZEL (*n* = 677)**		**Whitehall (*n* = 191)**	
	**OR** *	**95% CI**	**OR** *	**95% CI**	**OR** *	**95% CI**
Age (years)						
<50	1.00		1.00		1.00	
≥50	1.75	(1.12–2.74)	1.34	(0.99–1.82)	1.44	(0.63–3.32)
Occupational grade						
High	1.00		1.00		1.00	
Intermediate /low	1.11	(0.70–1.77)	1.81	(1.31–2.49)	2.34	(0.77–7.16)
Married/ cohabited						
Yes	1.00		1.00		1.00	
No	1.24	(0.73–2.11)	0.92	(0.56–1.51)	1.25	(0.48–3.22)
	**Women**					
	**FPSS (*n* = 947)**		**GAZEL (*n* = 163)**		**Whitehall II (*n* = 80)**	
	**OR** *	**95% CI**	**OR** *	**95% CI**	**OR** *	**95% CI**
Age (years)						
<50	1.00		1.00		1.00	
≥50	2.21	(1.64–2.98)	0.80	(0.39–1.65)	1.76	(0.69–4.49)
Occupational grade						
High	1.00		1.00		1.00	
Intermediate /low	1.64	(1.12–2.39)	2.00	(0.75–5.31)	0.93	(0.15–5.61)
Married/ cohabited						
Yes	1.00		1.00		1.00	
No	1.16	(0.84–1.61)	0.90	(0.41–2.00)	1.40	(0.55–3.59)
	**Pooled estimate from meta-analyses**					
	**Men (*n* = 1,229)**		**Women (*n* = 1,190)**		**All (*n* = 2,419)**	
	**OR** *	**95% CI**	**OR** *	**95% CI**	**OR** *	**95% CI**
Age (years)						
<50	1.00		1.00		1.00	
≥50	1.46	(1.14–1.85)	1.89	(1.45–2.47)	1.64	(1.37–1.96)
	I^2^ = 0.0%, *P* = 0.62		I^2^ = 69.4%, *P* = 0.04		I^2^ = 47.5%, *P* = 0.09	
Occupational grade						
High	1.00		1.00		1.00	
Intermediate /low	1.58	(1.22–2.04)	1.65	(1.16–2.33)	1.60	(1.30–1.97)
	I^2^ = 41.0%, *P* = 0.18		I^2^ = 0.0%, *P* = 0.77		I^2^ = 0.0%, *P* = 0.56	
Married/ cohabited						
Yes	1.00		1.00		1.00	
No	1.08	(0.77–1.52)	1.14	(0.86–1.52)	1.12	(0.90–1.39)
	I^2^ = 0.0%, *P* = 0.69		I^2^ = 0.0%, *P* = 0.77		I^2^ = 0.0%, *P* = 0.93	

*Sociodemographic factors (age, occupational grade and marital status) are mutually adjusted.

**Fig 2:**
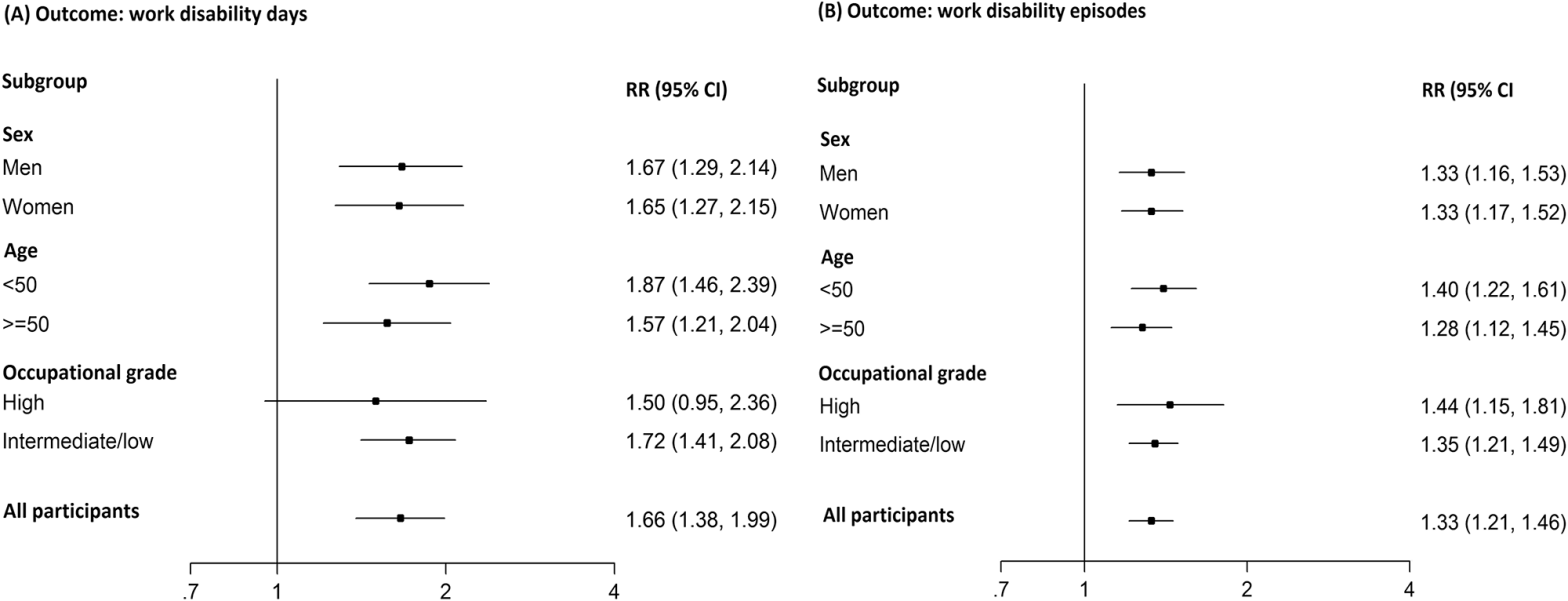
Adjusted pooled meta-analyses examining the association between membership of class 2 (‘high-risk profile’) compared to class 1 (‘low-risk profile’) and the number of work disability days (panel A) and episodes (panel B), in all participants of three study cohorts and in subgroups of sex, age and occupational grade.

Fig 2 presents summary estimates from fixed-effects meta-analyses of the association between class membership and work disability days (A) and episodes (B). Belonging to the ‘high-risk profile’ subgroup compared to ‘low-risk profile’ subgroup was associated with a 1.66 (95% CI 1.38–1.99) times higher rate ratio for work disability days and 1.33 (1.21–1.46) times higher rate ratio for the work disability episodes. Sub-group analyses indicated no difference in the association between men and women, age groups (≥50 years versus less), or occupational grade groups (all P-values for meta-regression >0.38).

Study-specific estimates for the association between ‘high-risk profile’ and work disability outcomes (S2 and S3 Figs) showed some heterogeneity in work disability days among women (I^2^ = 68.8%, P = 0.04) probably driven by a particularly strong association among women in GAZEL study. Heterogeneity in the overall estimate for work disability days was I^2^ = 41.3%, P = 0.13. In analysis of work disability episodes, no significant heterogeneity was found between cohorts (overall I^2^ = 0.0%, P = 0.54).

## Discussion

In studies of work disability, persons with diabetes have usually been treated as a single group without considering potential heterogeneity within the population with diabetes. Our findings suggest that there are two distinct subgroups of employees with diabetes, characterized by different prevalence of comorbid somatic diseases, psychological symptoms, obesity, physical inactivity and alcohol use. These two groups had different risk of future work disability.

There were two important observations in our study. First, in our analyses of single risk factors, there was, as expected, a strong association between prevalent comorbid somatic disease and psychological morbidity, and the number of work disability days and episodes. Of the health behaviours, obesity and low physical activity were associated with higher work disability days and episodes, while there was no association between smoking and these outcomes. These findings are partially in agreement with previous studies which have shown associations of obesity, low physical activity and smoking with sickness absence and disability pension in general working populations [8, 9, 12, 13]. For alcohol use, abstinence from alcohol (but not high alcohol use) was associated with higher number of work disability days and episodes, when compared to drinking alcohol within the recommended limits. This finding should be interpreted with caution because self-reported data may not accurately detect respondents with risky alcohol consumption. However, in previous studies, there is evidence that both abstinence of alcohol and risky drinking may be associated with an increased risk of work disability [8–11] although some studies have reported no association for risky drinking [11, 13].

Our second important observation was the identification of two latent classes, differentiated by those same risk factors we found to be associated with higher levels of work disability in our analyses of single risk factors: comorbid somatic disease, psychological symptoms, obesity, low physical activity and abstinence from alcohol. The only exception to this pattern was among the men and women in Whitehall II, with comorbid somatic disease among men and psychological symptoms among women clustering into different class than other risk factors, but due to a small sample sizes, particularly among women (n = 83), these may be due to chance only. In general, we observed that comorbid somatic disease, psychological symptoms, obesity, low physical activity and abstinence of alcohol appear to cluster in the same ‘high-risk profile’ subgroup of employees with diabetes. There was also a strong and consistent association between membership of this ‘high-risk profile’ subgroup and the number of future work disability days and episodes which were observed in men and women and across age and occupational grade groups.

We indexed work disability by two measures, the number of work disability days and episodes. Belonging to a ‘high-risk profile’ subgroup was more strongly associated with work disability days (RR 1.66, 95% CI 1.38–1.99) than episodes (RR 1.33, 95% CI 1.21–1.46), in line with the hypothesis that number of work disability days (i.e., absence duration) is primarily related to actual illness whereas the number of episodes (i.e., absence frequency) may also reflect motivational variables and withdrawal from adverse working conditions [30].

Participants who were older and from the lower occupational grades were more likely to be members of the ‘high-risk profile’ subgroup than younger participants and those with high occupational grade. Reasons to this were not possible to examine in our study although we also found that occupational grade was a very strong predictor of work disability. Association between low occupational grade and work disability might relate for example, to severity of diabetes and comorbid diseases, access to and quality of care, underutilisation of treatment, and adherence to treatment [31, 32].

There are limitations to this study. Diabetes was defined using the gold standard method, the 2-hour oral glucose tolerance test (in addition to self-report and medication) in the Whitehall II study only (phases 3 and 5). In GAZEL, diabetes was measured by self-report and in FPSS it was measured using medical records. Although the validity of self-reports and medical records of diabetes has been shown to be reasonably good [33], neither of the methods detect undiagnosed diabetes. Although a specific strength of our study is individual, daily-based register data on work disability measured as sickness absence and disability pension, in FPSS, sickness absence registers covered absences that lasted over 9 days only and in Whitehall II, sickness absence was register-based whereas work disability pension was based on survey responses.

There were also some differences between studies in the measurement of risk factors. Furthermore, although we assessed several important risk factors of work disability in people with diabetes, we were unable to examine other features, such as duration and severity of diabetes, quality of diet, and adherence to treatment, which might also be important. Although we sought to select comorbid conditions based on previous findings on their importance in diabetes prognosis and their importance in predicting work disability, we were limited by the availability of these measures in all three cohorts. Some diabetes-specific comorbid conditions, such as retinopathy, neuropathy and nephropathy, would be important to include in future studies. In our data, abstainers from alcohol included both never and former users, some of whom might abstain from alcohol due to health reasons or former problems with heavy drinking. Distinction of these groups among abstainers should be made in future studies to increase understanding of the impact of abstinence. In line with our findings, current clinical guidelines for treating diabetes emphasise tackling comorbidity, obesity, and low physical activity, in addition to diet, to treat and manage diabetes [34].

As in all observational studies, we cannot rule out the possibility of other unknown or unmeasured confounders or reverse causation, and due to the specific occupational samples, the results are not generalizable to wider working populations. Other specific strengths of our study are the use of three independent cohorts with relatively large number of participants with diabetes and prospective study design, and a meta-analytic approach which takes into account the variance between study cohorts.

Although LCA has previously been applied to research in several medical disciplines, including pulmonology [35] and psychiatry [36], to our knowledge it has not previously been applied to studies of diabetes and work disability. A major advantage of LCA is that it is an effective method to condense data. Instead of performing single analyses for each risk factor and all outcomes, as we did in Fig 1, LCA reduced the data into two distinct classes which in this case practically told the same story but added evidence that the risk factors of work disability may be clustered into the same response profile—comorbid somatic and psychological morbidity, obesity, low physical activity and abstinence from alcohol. In addition, a class-based analysis can guide stratified intervention strategies based on classes identified by LCA to reduce work disability among people with diabetes [37]. However, while LCA can suggest a certain number of discrete latent classes in observed data, it cannot prove that such discrete classes actually exist. The usefulness of the present classification is best assessed through validation and replication studies. Therefore, further studies are needed to examine whether the present findings are replicated in other cohorts and study settings and whether interventions based on latent classes are effective in prevention of work disability.

## Supporting Information

S1 Fig Adjusted pooled meta-analyses examining the association of single sociodemographic and health-related factors with number of work disability days and episodes in three study cohorts. (TIF)

S2 Fig Adjusted meta-analysis of the association between ‘high-risk profile’ and the number of work disability days among men and women in three study cohorts. (TIF)

S3 Fig Adjusted meta-analysis of the association between ‘high-risk profile’ and the number of work disability episodes among men and women in three study cohorts. (TIF)

S1 Table Model comparisons and fit indices in the three cohort studies; Finnish Public Sector, GAZEL and Whitehall II. (DOCX)
