# Supplementary figures and images for: Work Disability among Employees with Diabetes: Latent Class Analysis of Risk Factors in Three Prospective Cohort Studies
Source: PLoS One. 2015 Nov 16;10(11):e0143184. doi: 10.1371/journal.pone.0143184 (PMC4646666; doi:10.1371/journal.pone.0143184)

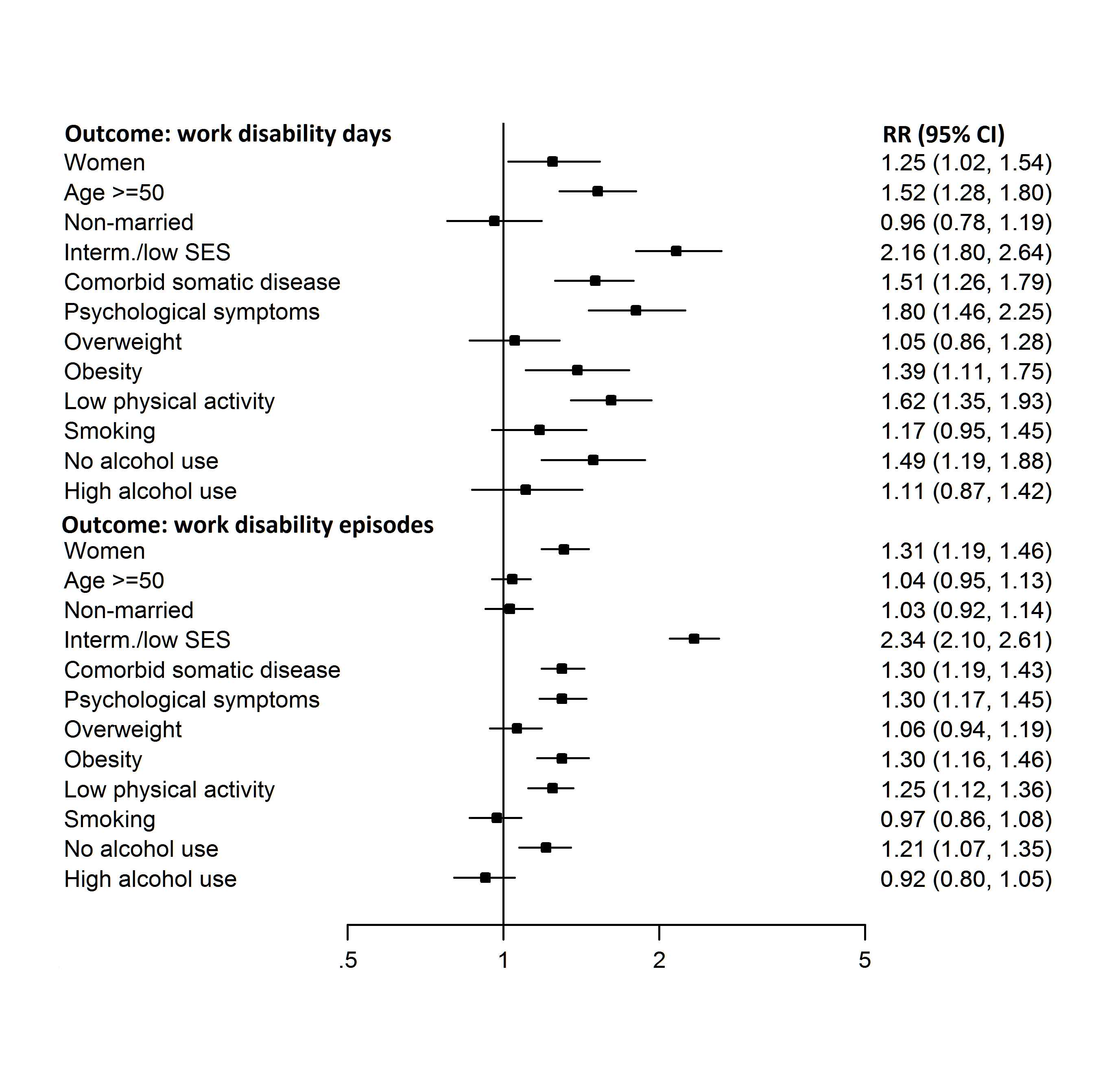

Supplement: S1 Fig — (TIF) [file pone.0143184.s001.tif]

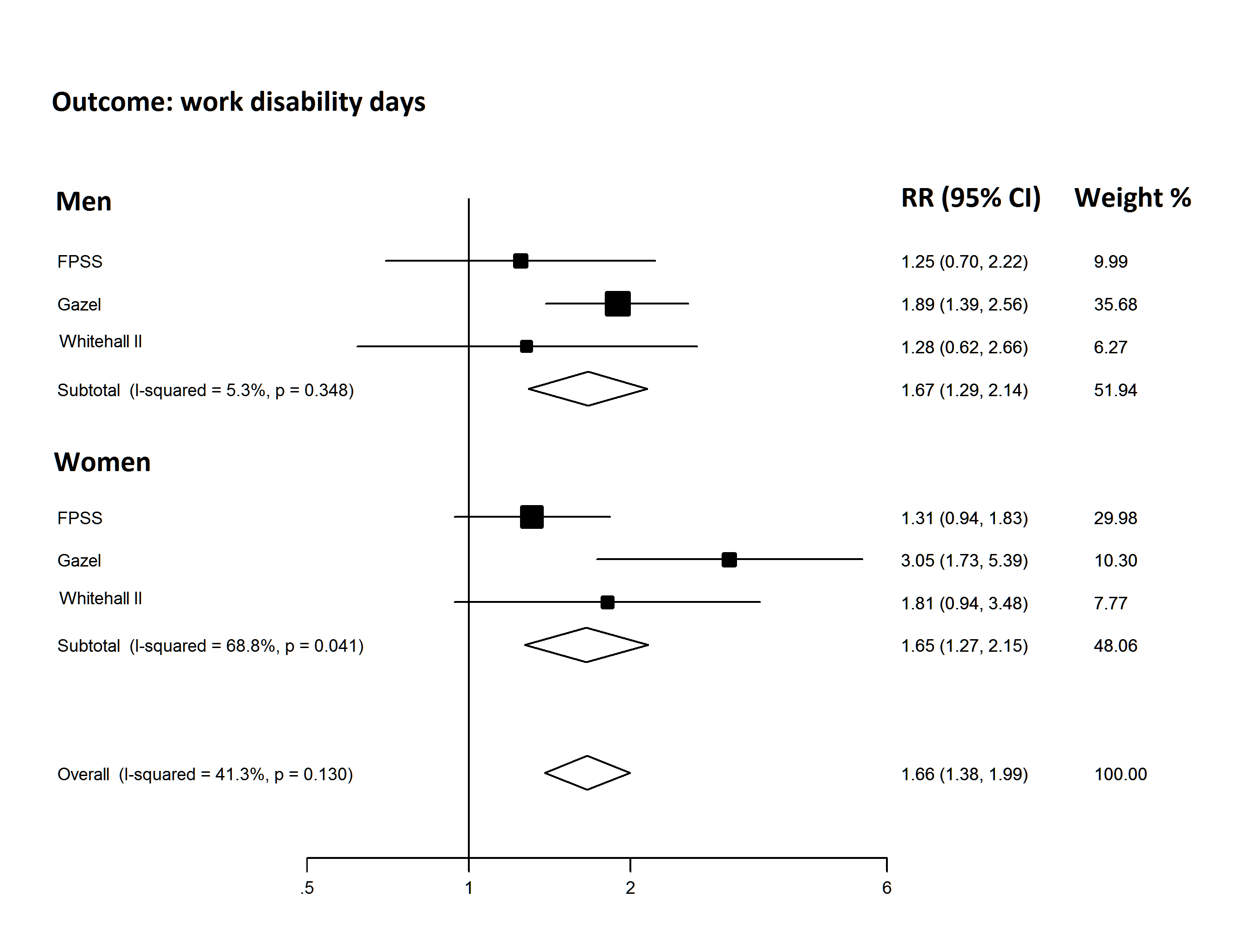

Supplement: S2 Fig — (TIF) [file pone.0143184.s002.tif]

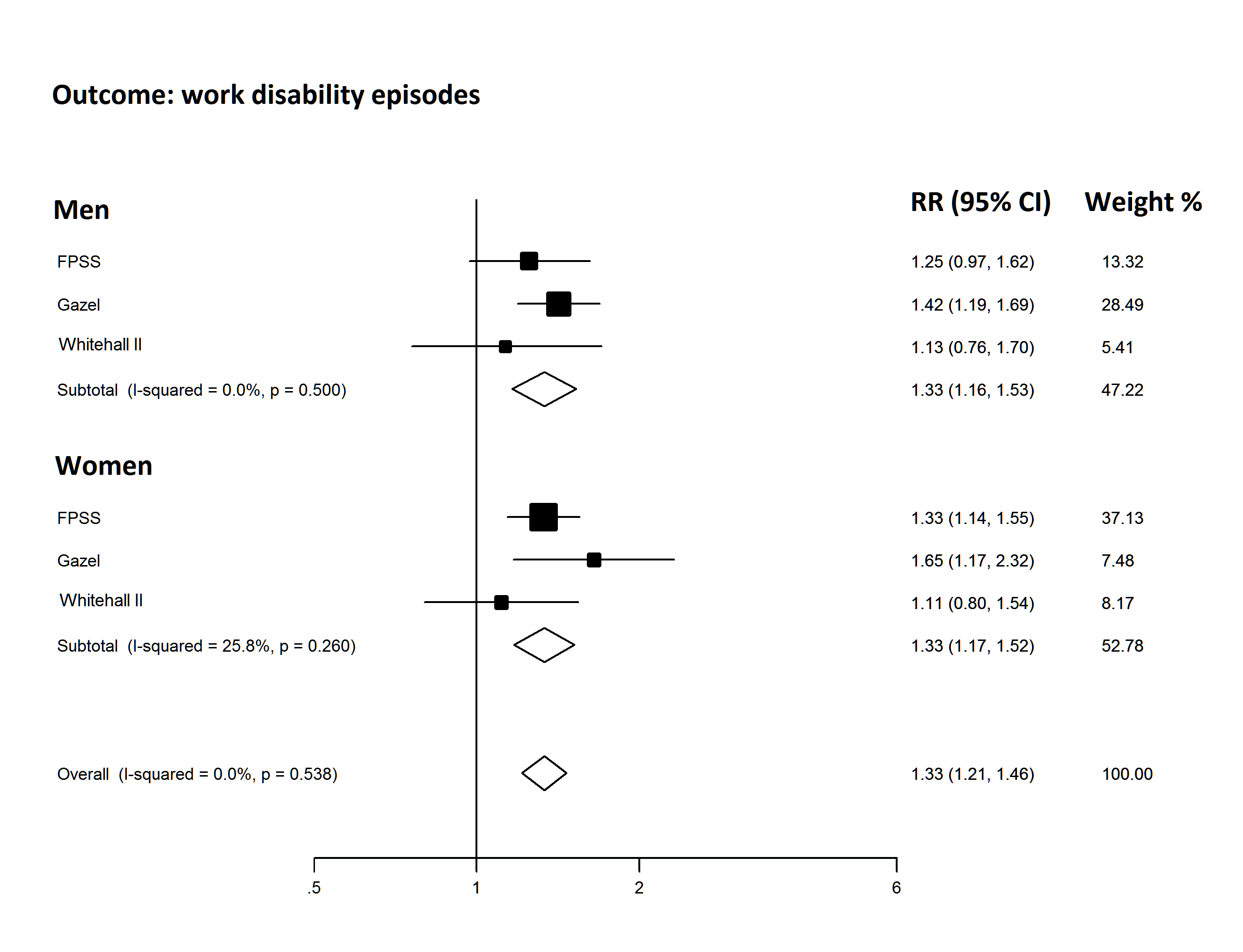

Supplement: S3 Fig — (TIF) [file pone.0143184.s003.tif]
