# Supplementary material for: Work Disability among Employees with Diabetes: Latent Class Analysis of Risk Factors in Three Prospective Cohort Studies
Source: PLoS One. 2015 Nov 16;10(11):e0143184. doi: 10.1371/journal.pone.0143184 (PMC4646666; doi:10.1371/journal.pone.0143184)
Supplement: S1 Table — (DOCX) [file pone.0143184.s004.docx]

**S1 Table.** Model comparisons and fit indices in the three cohort studies; Finnish Public Sector, GAZEL and Whitehall II.

|  | **AIC** | **BIC** | **CAIC** | **Adjusted BIC** | **Entropy** |
| --- | --- | --- | --- | --- | --- |
| **Finnish Public Sector study** |  |  |  |  |  |
| 2 classes | 158.3407 | 236.1669 | 251.1669 | 188.5187 | 0.463528 |
| 3 classes | 128.5078 | 247.8413 | 270.8413 | 174.7808 | 0.523598 |
| 4 classes | 120.2861 | 281.1269 | 312.1269 | 182.6540 | 0.511792 |
| 5 classes | 126.7182 | 329.0663 | 368.0663 | 205.1810 | 0.627807 |
| 6 classes | 135.6448 | 379.5002 | 426.5002 | 230.2026 | 0.588432 |
| 7 classes | 140.6685 | 426.0312 | 481.0312 | 251.3212 | 0.589980 |
| 8 classes | 152.4582 | 479.3282 | 542.3282 | 279.2059 | 0.623015 |
| 9 classes | 162.9430 | 531.3203 | 602.3203 | 305.7857 | 0.569016 |
| **GAZEL study** |  |  |  |  |  |
| 2 classes | 131.9810 | 203.0177 | 218.0177 | 155.3825 | 0.369773 |
| 3 classes | 130.4102 | 239.3331 | 262.3331 | 166.2925 | 0.494530 |
| 4 classes | 132.9139 | 279.7231 | 310.7231 | 181.2769 | 0.449241 |
| 5 classes | 137.3567 | 322.0521 | 361.0521 | 198.2005 | 0.530679 |
| 6 classes | 143.2426 | 365.8242 | 412.8242 | 216.5672 | 0.575323 |
| 7 classes | 150.1218 | 410.5897 | 465.5897 | 235.9272 | 0.642949 |
| 8 classes | 161.3727 | 459.7268 | 522.7268 | 259.6589 | 0.589045 |
| 9 classes | 171.4699 | 507.7103 | 578.7103 | 282.2369 | 0.607578 |
| **Whitehall II study** |  |  |  |  |  |
| 2 classes | 111.0100 | 165.4782 | 180.4782 | 117.9145 | 0.404014 |
| 3 classes | 107.5154 | 191.0333 | 214.0333 | 118.1023 | 0.505319 |
| 4 classes | 114.1764 | 226.7439 | 257.7439 | 128.4457 | 0.580146 |
| 5 classes | 122.1988 | 263.8161 | 302.8161 | 140.1506 | 0.621721 |
| 6 classes | 130.4387 | 301.1057 | 348.1057 | 152.0729 | 0.665980 |
| 7 classes | 138.7233 | 338.4400 | 393.4400 | 164.0399 | 0.697829 |
| 8 classes | 148.8899 | 377.6562 | 440.6562 | 177.8888 | 0.692542 |
| 9 classes | 158.4891 | 416.3051 | 487.3051 | 191.1705 | 0.764490 |

Note. Model comparison measurements for choosing the optimal number of classes in latent class analysis (the models with the smallest values indicate a better fit). AIC (Akaike's information criterion), BIC (Bayesian information criterion), CAIC (consistent AIC), adjusted BIC and entropy; all measure the model fit.
